# Mechanistic Investigation of ROS-Induced DNA Damage by Oestrogenic Compounds in Lymphocytes and Sperm Using the Comet Assay

**DOI:** 10.3390/ijms12052783

**Published:** 2011-04-28

**Authors:** Eduardo Cemeli, Diana Anderson

**Affiliations:** Genetic and Reproductive Toxicology Group, Division of Biomedical Sciences, University of Bradford, Bradford, West Yorkshire, BD7 1DP, UK; E-Mail: Eduardo_Cemeli@bat.com

**Keywords:** oestrogens, comet assay, lymphocytes, sperm, ROS, lipid peroxidation

## Abstract

Past research has demonstrated that oestrogenic compounds produce strand breaks in the DNA of sperm and lymphocytes via reactive oxygen species (ROS). In the current investigation, sperm and lymphocytes were treated *in vitro* with oestrogenic compounds (diethylstilboestrol, progesterone, 17β-oestradiol, noradrenaline and triiodotyronine) and several aspects of DNA damage were investigated. Firstly, mediation of DNA damage by lipid peroxidation was investigated in the presence of BHA (a lipid peroxidation blocker). BHA reduced the DNA damage generated by 17β-oestradiol and diethylstilboestrol in a statistically significant manner. No effects were observed for sperm. Secondly, the presence of oxidized bases employing FPG and EndoIII were detected for lymphocytes and sperm in the negative control and after 24 h recovery in lymphocytes but not immediately after treatment for both cell types. The successful detection of oxidized bases in the negative control (untreated) of sperm provides an opportunity for its application in biomonitoring studies. DNA repair at 24 h after exposure was also studied. A nearly complete recovery to negative control levels was shown in lymphocytes 24 h recovery after oestrogenic exposure and this was statistically significant in all cases. Rapid rejoining of DNA, in a matter of hours, is a characteristic of DNA damaged by ROS.

## 1. Introduction

Oestrogens play a key role in the development of feminine characteristics, control of reproductive cycles and pregnancy, and also have an influence on the skin, bone, the cardiovascular system and immunity [1]. In recent years, exposure to oestrogens and oestrogen-like compounds has been associated with the development of cancer and other effects [2]. A strong positive association between breast cancer risk and circulating levels of both oestrogens and androgens has been confirmed [3,4]. Epidemiological data implicate an imbalance of oestrogens and progestogens in the etiology of endometrial cancer [5]. In particular, the Women’s Health Initiative Study demonstrated that women receiving hormone replacement therapy (HRT), consisting of oestrogen plus progestin, have an increased risk of endometrial and invasive breast carcinoma [6]. Steroid hormones are involved in ovarian carcinogenesis [7,8] and a possible relationship exists between sex steroids and colon cancer [7,9]. Oestrogens have been implicated in the development of prostate cancer [10,11]. Moreover, they may also affect male fertility since xenoestrogens have been flagged as major culprits of male reproductive abnormalities [12]. The WHO-IARC classified oestrogens, steroidal and non-steroidal, as carcinogenic in humans [13], one of the main arguments being the fact that oestrogens not only can promote cancers but also may initiate mutations caused by certain oestrogen metabolites [14]. Their mechanisms of action are varied, including stimulation of cellular proliferation through receptor-mediated hormonal activity, increasing genetic mutation rates through cytochrome P450-mediated metabolic activation, and induction of aneuploidy [15].

Our group found evidence of the involvement of reactive oxygen species (ROS) in the DNA damage generated by phytoestrogens and oestrogenic compounds when investigated by means of the Comet assay [16,17]. Likewise, a later investigation stated that oxidative stress resulting from metabolic activation of carcinogenic oestrogens plays a critical role in oestrogen-induced carcinogenesis as confirmed in experiments in hamsters [18]. Oxidized metabolites from 17β-oestradiol have been proved to elicit cytotoxicity in cultured human mammary epithelial cells, which was blocked by the antioxidant trolox [19]. The interaction of catechol-oestrogen and copper leads to the production of ROS which eventually cause DNA damage [20]. Recently, it has been reported that, in addition to the direct effect of oestrogens on mitochondria and the redox cycling of catechol-oestrogen, oestrogen-induced pro-inflammatory cytokines, such as interleukin-1β and tumor necrosis factor α, also generate reactive oxygen and nitrogen species (RO/NS) [21]. Endogenous non-steroidal hormones and related compounds such as neurotransmitters are present at low concentrations in the body, regulate physiological processes and have stimulatory effects on metabolic activities [22]. The thyroid hormones appear to regulate the duration of Sertoli cell proliferation, affecting adult Sertoli cell number and therefore the capacity to produce sperm [23]. In the thyroid, ROS and free radicals participate in physiological and pathological processes in the gland [24]. Also, during the normal catabolic pathway of oxidation of noradrenaline, ROS and H_2_O_2_ are produced [25] and this might be the cause for the genotoxicity reported for thyroid hormones and noradrenaline [26].

In the present investigation, we aimed to determine a number of characteristics linked to the presence of ROS. These were: (1) mediation of DNA damage through lipid peroxidation employing butylated hydroxyanisol (BHA); (2) detection of the presence of oxidized DNA bases employing specialized enzymes: formamidopyrimidine DNA Glycosylase (FPG) and endonuclease III (endo III); and (3) DNA repair at a single time point of 24 h. The cells selected for this investigation are of relevance for different reasons. Lymphocytes are commonly employed as surrogate cells whilst sperm have been demonstrated to be affected by oestrogens. Furthermore, we investigated the described characteristics on five oestrogenic compounds (diethylstilboestrol (DES), progesterone (PG), 17β-oestradiol, noradrenaline (NA) and triiodotyronine (T3)) providing a wide variety of functions and molecular structures to this study. We understand that the effects generated by oestrogens occur at lower concentrations. Physiological concentrations of these compounds are typically in the picoM to nM range: 17β-oestradiol (0.1 to 1.6 nM) [27], PG (30 to 600 nM) [28], NA (2 to 4 nM) [29] and T3 (1 to 3 nM) [30]. However, exposure occurs over longer periods of time and such conditions may become difficult to reproduce *in vitro* in a laboratory. Furthermore, we see a need to generate effects in order to be able to reveal the mechanisms underlying them. The concentrations selected have already been employed in previous investigations [16,17,22,26,31].

## 2. Results and Discussion

### 2.1. Mediation of Lipid Peroxidation in the Generation of DNA Damage

Lipid peroxidation appears to be a major source of endogenous DNA damage in humans that may contribute significantly to cancer and other genetic diseases linked to lifestyle and dietary factors [32]. ROS have been reported to impair membrane function [33]. This occurs in an initiation stage through the extraction of hydrogen from membrane lipids by ROS such as the hydroxyl radical (HO) [34]. Peroxyl radicals (ROO) are formed in the propagation stage, which after a cascade of reactions, ends up yielding deleterious effects to DNA. In sperm, oxidative stress is known to play a major role in the etiology of defective sperm function via mechanisms involving the induction of peroxidative damage to the plasma membrane [35].

Human PBL and sperm were co-incubated with oestrogenic compounds in the presence and absence of butylated hydroxyanisol (BHA). BHA is a well-known lipid peroxidation blocker used in the food industry [36]. This was in order to find out whether its presence modulated DNA damage by reducing or abolishing lipid peroxidation generated by oestrogens. By halting the lipid peroxidation, the end products are impeded in reacting with the DNA molecule. BHA at a concentration of 500 μM was selected on the basis of its DNA protective effects to a ROS inducing agent in CHO cells [37]. Reduction in genotoxic effects has also been observed in another investigation at this concentration [38].

**Figure 1.**
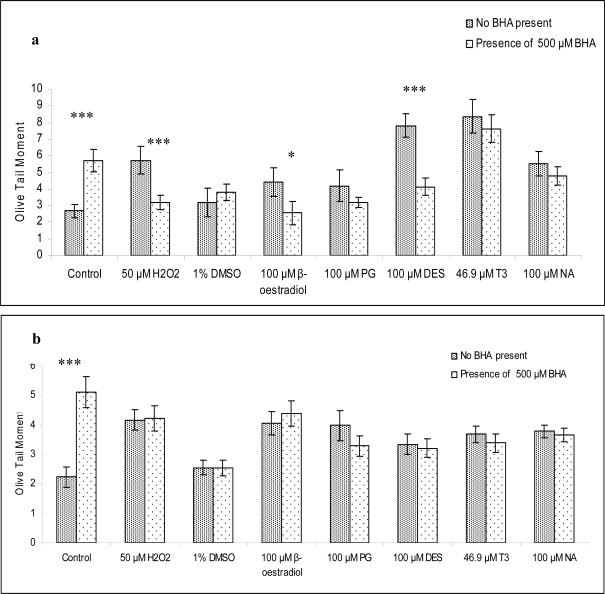
The Olive Tail Moment values for lymphocytes **(a)** and sperm **(b)** treated with oestrogenic compounds or H_2_O_2_ for 30 min at 37 °C in the presence and absence of BHA (500 μM). The figures express the mean of the values obtained from 7 independent experiments (n = 7 different donors) in (a) and 5 independent experiments (n = 5 different donors) in (b). For (a) the average age was 27.57 ± 2.41 and for (b) the average age was 30.80 ± 2.35. Normality of the data was evaluated with the Shapiro-Wilks test. Data were normally distributed. The Student’s T-test was applied. For statistical interpretation of the data, treatment in the absence of BHA was compared to treatment with the same compound with its presence. The levels of significance are included: n.s. not significant; *p ≤ 0.05; **p ≤ 0.01 and ***p ≤ 0.001.

In Figure 1a and b, the effect of BHA on the genotoxicity of oestrogenic compounds on lymphocytes and sperm is displayed. The negative control for lymphocytes and sperm in the absence of BHA provided an Olive tail moment value which was within the historical records in our laboratory as observed in publications by this group. BHA at a concentration of 500 μM exhibited a pro-oxidant effect which generated DNA damage in a statistically significant manner in both cell types. All oestrogenic compounds, the solvent (DMSO) and the positive control (H_2_O_2_) induced an increase in the values of Olive tail moment when compared to the negative control in both cell types. In lymphocytes (Figure 1a), BHA reduced the DNA damage generated by the oestrogenic compounds and H_2_O_2_ in all cases with the exception of DMSO. The reduction was statistically significant in some instances. By contrast, no reduction by BHA was observed at any treatment in sperm.

BHA on its own produced a pro-oxidant response in both lymphocytes and sperm. Genotoxic effects of BHA were already observed in gastrointestinal organs from mouse when measured with the Comet assay [39]. The presence of BHA reduced the levels of DNA damage in lymphocytes in all oestrogenic treatments and it abolished strand breaks against H_2_O_2_. Thus, this may indicate that DNA damage induced by oestrogenic compounds in lymphocytes is mediated, at least partly, through lipid peroxidation. This could not be demonstrated for sperm since the responses obtained in the presence of BHA did not show a clear and common pattern. This poses an interesting outcome since sperm are known to possess a high content of PUFA. It might be possible that 500 μM BHA is not sufficient to counteract the genotoxic effects of the oestrogenic compounds in sperm. Further work is necessary to clarify the described effect in sperm.

### 2.2. Detection of Oxidized Bases Induced by Oestrogenic Compounds in Lymphocytes and Sperm

In addition to the endocrine-disrupting capability of oestrogenic compounds, further disruptive effects have been reported when oestrogenic compounds are metabolized. ROS and oestrogenic metabolites are produced which readily interact with the DNA structure and compromise its integrity [21]. In this regard, a report affirms that oestrogenic compounds can have a powerful impact on human spermatozoa via the induction of redox cycling behavior and the creation of adducts that cross-link DNA [40]. Therefore, we understand that there is evidence supporting that potentially oxidized bases are produced. With regard to the detection of oxidized bases, the Comet assay has been demonstrated to be the most sensitive and accurate technique for measurement of oxidized bases since other techniques have proved to be prone to generation of artifacts [41]. For this reason, Endo III and FPG were selected to identify oxidized bases in the Comet assay. The treatment period was also extended in order to increase the DNA damaging effects and; in turn, allow a more clear reflection of DNA repair in case it occurred. Cell viability was ensured after extended cell treatment.

In Table 1, the data obtained for the detection of oxidized bases induced by oestrogenic treatment in lymphocytes as well as sperm are shown. The presence of enzymatic buffer in the negative control of both cell types provided values within historical records in our laboratory as observed in publications by this group. In the negative control corresponding to the experiment of lymphocytes without repair, the presence of the enzymes FPG and EndoIII generated a statistically significant increase in the Olive tail moment values. A small but not statistically significant increase was observed for the repair control in presence of FPG and EndoIII. No statistically significant increases were observed for the detection of oxidized bases after treatment with FPG or EndoIII in the presence or absence of repair. Nevertheless, detection of oxidized bases proved more consistent after repair since all treatments displayed values over their corresponding control levels. With regard to sperm, the presence of FPG and Endo III generated a statistically significant increase in the Olive tail moment of the negative control. No responses or even small reductions were produced in the oestrogenic treatments in the presence of FPG and EndoIII.

**Figure 2.**
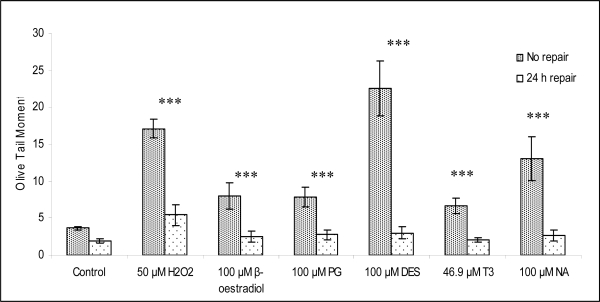
The Olive Tail Moment values for fresh lymphocytes treated with oestrogenic compounds or H_2_O_2_ for 120 min and after 24 h repair in fresh medium. The mean of the values obtained from 7 independent experiments are shown (n = 7 different donors). Average age is 27.57 ± 2.41. Normality of the data was evaluated with the Shapiro-Wilks test. Data were normally distributed. The Student’s T-test was applied. For statistical interpretation of the data, no repair *versus* repair for each of the oestrogenic compounds was compared. The levels of significance are included: n.s. not significant; *p ≤ 0.05; **p ≤ 0.01 and ***p ≤ 0.001.

There was consistency amongst all oestrogenic compounds in the detection of oxidized bases after 24 h repair in lymphocytes but they were not statistically significant. It is known that a critical step in the detection of oxidized bases after exposure to genotoxicants is the need for a recovery period in order to repair the strand breaks generated leaving the oxidized bases exposed which will be cleaved by FPG and EndoIII. The selection of a repair time which is too short results in confusion between strand breaks produced by the investigated genotoxicant and the ones originated by the enzymes (FPG ad EndoIII). The fact that the DNA repair period reduced the Olive tail moment to nearly negative control levels (Figure 2) for all the treatments confirms that 24 h repair is an adequate recovery period. However, the possibility cannot be ruled out that a slightly longer repair, bringing Olive tail moment values down to negative control levels, might allow a more optimized detection of the oxidized bases.

Two investigations have provided evidence of the capability of oestrogenic compounds to induce oxidized bases. Endo III, but not FPG, enhanced the DNA damaging effects of catechol in mouse lymphoma cells and extended term human lymphocytes [42]. FPG recognized oxidized bases for 17β-oestradiol, 2-OH and 4-OH oestradiol in human breast cells MCF-7 [43]. The success of such investigations might be explained by a faster repair in cell lines compared to fresh lymphocytes. According to Collins, freshly isolated (as opposed to stimulated) lymphocytes rejoin strand breaks more slowly and this might reflect the need for lymphocytes to adapt to sudden exposure of oxygen in the atmosphere [44]. In this sense, the lack of repair in mature spermatozoa may be the reason for the absence of detection in treated sperm with high levels of DNA damage.

The detection of oxidized bases in the negative controls of lymphocytes should not be surprising since there are large numbers of publications employing these two enzymes for monitoring purposes in lymphocytes [45–47]. Therefore, our results further support the validity of such approaches. To date, only one publication has measured oxidized bases in human testicular cells [48] and, to our knowledge, none has for ejaculated spermatozoa. The data displayed in the current investigation demonstrates the feasibility for detecting oxidized bases in ejaculated sperm when levels of DNA damage are low. These results might further stimulate research based on monitoring males for oxidized bases in ejaculated sperm and the correlation of such bases to environmental exposure and reproductive outcomes.

### 2.3. DNA Repair after Oestrogenic Treatment

A third mechanistic aspect investigated in the present study was DNA repair at 24 h after exposure to oestrogenic compounds as shown in Figure 2. It is widely known that strand breaks induced by ROS are quickly repaired. For instance, after 5 min repair time following treatment with H_2_O_2_ approximately 30% of the DNA was repaired in well-nourished children and at 60 min this capacity increased to 82% [49]. The literature provides examples for other investigations with a similar outcome as it is the case of DNA repair in an *in vitro* study of nickel exposure [50] or repair in a cell lineage model of human proliferative breast disease [51]. To allow DNA repair to take place, thus reducing the levels of DNA strand breaks, an experimental design involving a recovery period was carried out in lymphocytes. This was not performed in sperm since mature spermatozoa are not able to repair DNA damage. The reason is due to a loss of cytoplasmic content during the maturation process [52–54].

Hence, blood was obtained from 7 subjects and lymphocytes were isolated immediately before the performance of the experiment. The recovery time was 24 h and it was applied after 120 min treatment. These times were selected in order to maximize the effects. A clear reduction in the levels of DNA damage was demonstrated for all treatments and also included the negative control. Such an outcome may add further evidence supporting the generation of oxidative stress by oestrogenic compounds.

## 3. Materials and Methods

### 3.1. Human PBL Isolation

Heparinized blood samples were obtained by venepuncture from 7 healthy male volunteers at the University of Bradford (UK) after signing a consent form. The procedures had been cleared by the University of Bradford’s Research Ethics sub-committee for Research involving Human subjects (Reference number: 0405/8). The average age was 27.57 ± 2.41 years old. All subjects were non-smokers and stated to be healthy. Lymphocytes were isolated from whole blood using Lymphoprep^®^ (Axis-Shield, Oslo, Norway) and the aliquots were stored in cryovials containing FBS with 1% DMSO for short-time storage at −80 °C. Freshly isolated lymphocytes were employed for the investigations detecting oxidized bases and DNA repair.

### 3.2. Human Semen Evaluation

Five subjects donated sperm. All semen samples were obtained with a signed consent form by the donor. The procedures had been cleared by the University of Bradford’s Research Ethics Sub-committee for Research involving Human subjects (Reference number: 0405/08). The average age was 30.8 ± 2.35 years old. All subjects were non-smokers and stated to be healthy. Semen samples were processed as described in the WHO criteria [13]. Semen was assessed shortly after ejaculation for the following parameters: viscosity, morphology, motility, pH and concentration. All samples provided parameters considered as normal. After evaluation, the ejaculate was aliquoted in cryovials, flash-frozen in liquid nitrogen and stored at −80 °C.

### 3.3. Comet Assay in Human PBL

Treatments with the oestrogenic compounds were carried out in PBS at 37 °C and the time of exposure varied depending on the experimental design. For the studies on lipid peroxidation, a co-treatment of 30 min including the oestrogenic compound and BHA was carried out. For the detection of oxidized bases, the treatment was 120 min. In addition, a 24 h recovery period was allowed for characterization of DNA repair. The oestrogenic compounds (17β-oestradiol [CAS; 50-28-2], DES [CAS; 56-53-1], noradrenaline [CAS; 586-17-4], progesterone [CAS; 57-83-0], triiodothyronine (T3) [CAS; 5817-39-0], H_2_O_2_ [CAS; 7722-84-1] and BHA [CAS; 25013-16-5]) were purchased from Sigma. Dose response studies were carried out for all the compounds investigated (data not shown). Viability was evaluated with trypan blue (0.4%) (Sigma) and fluoresceine diacetate (Sigma) and it was greater than 75% for the concentrations selected for this investigation [55]. The Comet assay was performed as recommended in the IWGT Comet assay guidelines [56].

Oxidative DNA damage was evaluated using the bacterial enzymes FPG and EndoIII kindly provided by Prof. Andrew Collins (University of Oslo, Norway). The cell line lymphoblastoid TK6 was employed to optimize the dilutions of FPG and endoIII to be used with sperm and lymphocytes. After overnight immersion in lysing buffer, the slides were rinsed and left covered with enzyme buffer containing a specific concentration of enzyme (1:3000) for both FPG and endoIII. The procedure was as described by Collins and co-workers [57,58]. After enzymatic incubation, the slides were rinsed again and placed in the electrophoresis tank. Thereafter, the procedure followed was as for the experiments in which no enzymes were used.

### 3.4. Comet Assay with Human Sperm

Sperm cryovials were quickly thawed. Treatment with oestrogenic compounds was carried out for 30 min for the lipid peroxidation investigation and 120 min for the oxidized bases. The treatment was at 32 °C which is the optimum temperature for sperm. After treatment, Eppendorf^®^ tubes were gently centrifuged and the supernatant was removed. Treated cells were embedded in 2% LMP agarose and left to set on ice for 5 min. A third agarose layer (0.5%) LMP was included for those experiments not requiring enzymatic treatment. The slides were left for another 5 minutes on ice after which they were immersed in a Coplin jar containing 10 mM DTT (Sigma) for 60 min at 4 °C. Afterwards, slides were rinsed in PBS and newly immersed in a Coplin jar containing 0.05 mg/mL proteinase K (Roche) for 60 min at 4 °C. If slides had to be treated with enzymes, an additional incubation with either FPG or EndoIII was carried out at this stage. Then, slides were rinsed again with excess electrophoresis buffer and placed in the electrophoresis tank. Unwinding lasted for 20 min followed by electrophoresis for another 20 min (25 V, 300 mA). Thereafter, the procedure carried out for the sperm was as previously described for lymphocytes.

### 3.5. Evaluation of the Data

Experiments were carried out independently and in duplicate in all investigations. Each experiment corresponded to a different subject for all investigations. All values in this study are expressed as the mean ± S.E.M. from 5 (sperm) or 7 (lymphocytes) independent experiments. Slides were coded before scoring and fifty cells from each duplicate were scored per concentration per experiment. The parameter selected for the measurement of DNA damage in lymphocytes and sperm was Olive tail moment [59]. With regard to the investigation for detecting oxidized bases, the mean Comet value for the control (buffer) was subtracted from the mean score of the control with FPG or Endo III. The values obtained were considered as the increase due to the detection of oxidized bases in the control (background oxidized bases). Such values were subtracted from the values obtained after treatment with compounds (H_2_O_2_, 17β-oestradiol, progesterone, DES, T3 and noradrenaline) and FPG or Endo III. This was in order to only consider the oxidized bases generated by the oestrogenic treatment and not those present in the background. The statistics package used was Statistica 6.1 (Tulsa, OK, USA). P values of less than 5% were considered significant.

## 4. Conclusion

The present study reveals some possible mechanisms involved in the genotoxicity of oestrogenic compounds. It indicates that lipid peroxidation may contribute to the generation of strand breaks in the DNA of lymphocytes whilst the concentration of BHA selected may not be appropriate for sperm. Consistent detection of oxidized bases, though to a minor extent, was obtained in lymphocytes after DNA repair. This was not observed in sperm and it is likely to be due to their lack of DNA repair ability. However, oxidized bases were detected in the negative control and such a finding has a vast potential in biomonitoring studies. Lastly, very clear evidence of DNA repair was found after exposure to oestrogenic compounds in lymphocytes. This is characteristic of DNA damaged by ROS. This characterization was performed in lymphocytes which are extensively used as surrogate cells and sperm which is a target for oestrogenic compounds. Interestingly, DNA in somatic cells (lymphocytes) and sperm present major differences: DNA in sperm being half the content as well as more tightly packed. Despite such differences, strand breaks induced by five oestrogenic compounds were produced in both cell types for all the compounds selected.

## Figures and Tables

**Table 1.** Treatment of lymphocytes and sperm with 5 oestrogenic compounds, H_2_O_2_ and the negative control with and without the presence of FPG (1:3000) and Endo III (1:3000).  The table expresses the mean of the values obtained from 7 independent experiments (n = 7 different donors) for lymphocytes and 5 independent experiments (n = 5 different donors) for sperm. For lymphocytes the average age was 27.57 ± 2.41. For sperm the average age was 30.8 ± 2.35. The data were evaluated for normality with the Shapiro-Wilks test. The data were normally distributed. ANOVA with Dunnet’s post-hoc was applied. For statistical interpretation of data, treatments with FPG or EndoIII were compared to the corresponding control.

**Compound**	**Treatment**	**Lymphocytes**		**Sperm**
		**120 min treatment**	**120 min followed by 24 h repair (Olive tail moment)**	**120 min (Olive tail moment)**
Control	Buffer	3.65 ± 0.28	1.89 ± 0.32	2.94 ± 0.24
	FPG	6.29 ± 0.85 (**)	2.34 ± 0.72 (n.s.)	3.96 ± 0.34 (**)
	EndoIII	6.36 ± 0.55 (**)	2.08 ± 1.65 (n.s.)	4.12 ± 0.36 (**)
H_2_O_2_	50 μM	17.13 ± 1.22	5.44 ± 1.36	4.98 ± 0.40
	FPG	16.72 ± 1.21 (n.s.)	6.51 ± 1.71 (n.s.)	4.91 ± 0.52 (n.s.)
	EndoIII	18.45 ± 1.16 (n.s.)	5.94 ± 1.80 (n.s.)	5.03 ± 0.47 (n.s.)
17 β-oestradiol	100 μM	8.01 ± 1.72	2.51 ± 0.70	5.52 ± 0.67
	FPG	8.89 ± 1.43 (n.s.)	2.97 ± 0.73 (n.s.)	6.24 ± 1.07 (n.s.)
	EndoIII	9.04 ±1.57 (n.s.)	3.01 ±0.65 (n.s.)	3.65 ± 0.50 (**)
Progesterone	100 μM	7.81 ± 1.34	2.76 ± 0.64	6.35 ± 0.57
	FPG	7.82 ± 1.23 (n.s.)	2.89 ± 0.63 (n.s.)	5.44 ± 0.65 (n.s.)
	EndoIII	7.04 ±1.09 (n.s.)	2.94 ±0.53 (n.s.)	5.82 ± 0.92 (n.s.)
DES	100 μM	22.62 ± 3.73	3.03 ± 0.86	6.55 ± 0.81
	FPG	24.90 ± 4.22 (n.s.)	4.80 ± 1.30 (n.s.)	5.48 ± 0.56 (n.s.)
	EndoIII	24.01 ± 3.24 (n.s.)	3.28 ± 1.56 (n.s.)	4.92 ± 0.75 (n.s.)
T3	46.9 μM	6.63 ± 1.03	2.02 ± 0.29	4.11 ± 1.12
	FPG	6.99 ± 1.31 (n.s.)	2.53 ± 0.33 (n.s.)	4.12 ± 0.43 (n.s.)
	EndoIII	6.09 ± 0.94 (n.s.)	2.28 ± 0.26 (n.s.)	4.45 ± 0.89 (n.s.)
Noradrenaline	100 μM	13.08 ± 2.91	2.72 ± 0.75	3.39 ± 0.70
	FPG	13.04 ± 1.30 (n.s.)	3.93 ± 1.23 (n.s.)	3.25 ± 0.77 (n.s.)
	EndoIII	13.50 ± 1.14 (n.s.)	2.78 ± 0.57 (n.s.)	2.79 ± 0.34 (n.s.)

The levels of significance are included: n.s. not significant;

* p ≤ 0.05;

** p ≤ 0.01 and

*** p ≤ 0.001.  H_2_O_2_: hydrogen peroxide; DES: diethylstilboestrol; T3: triiodo-L-thyronine.
